# Tetramethylpyrazine Nitrone (TBN) Reduces Amyloid β Deposition in Alzheimer’s Disease Models by Modulating APP Expression, BACE1 Activity, and Autophagy Pathways

**DOI:** 10.3390/ph17081005

**Published:** 2024-07-30

**Authors:** Xinhua Zhou, Zeyu Zhu, Shaoming Kuang, Kaipeng Huang, Yueping Li, Yuqiang Wang, Haiyun Chen, Maggie Pui Man Hoi, Benhong Xu, Xifei Yang, Zaijun Zhang

**Affiliations:** 1 Guangzhou Medical Research Institute of Infectious Diseases, Guangzhou Eighth People’s Hospital, Guangzhou Medical University, Guangzhou 510440, China; xinhuazhou9901@163.com (X.Z.); kshming@foxmail.com (S.K.); gz8hhkp@126.com (K.H.); gz8hlypicu@126.com (Y.L.); 2 State Key Laboratory of Bioactive Molecules and Druggability Assessment, Guangzhou Key Laboratory of Innovative Chemical Drug Research in Cardio-Cerebrovascular Diseases, Institute of New Drug Research, Jinan University, Guangzhou 511436, China; zhuzy23@mail3.sysu.edu.cn (Z.Z.); yuqiangwangjnu@163.com (Y.W.); 3 Guangdong-Hong Kong-Macau Joint Laboratory for Pharmacodynamic Constituents of TCM and New Drugs Research, Guangdong Province Key Laboratory of Pharmacodynamic Constituents of TCM and New Drugs Research, Jinan University College of Pharmacy, Guangzhou 511436, China; 4 International Cooperative Laboratory of Traditional Chinese Medicine Modernization and Innovative Drug Development of Chinese Ministry of Education (MOE), Jinan University College of Pharmacy, Guangzhou 511436, China; 5 School of Pharmacy, Guangdong Pharmaceutical University, Guangzhou 510006, China; yx07230chy@126.com; 6 State Key Laboratory of Quality Research in Chinese Medicine, Institute of Chinse Medical Sciences, University of Macau, Macau, China; maghoi@um.edu.mo; 7 Key Laboratory of Modern Toxicology of Shenzhen, Shenzhen Center for Disease Control and Prevention, Shenzhen 518055, China; xubenhong0916@163.com

**Keywords:** amyloid β, amyloid precursor protein, microRNA

## Abstract

Alzheimer’s disease (AD) is a neurodegenerative disorder associated with age. A wealth of evidence indicates that the amyloid β (Aβ) aggregates result from dyshomeostasis between Aβ production and clearance, which plays a pivotal role in the pathogenesis of AD. Consequently, therapies targeting Aβ reduction represent a promising strategy for AD intervention. Tetramethylpyrazine nitrone (TBN) is a novel tetramethylpyrazine derivative with potential for the treatment of AD. Previously, we demonstrated that TBN markedly enhanced cognitive functions and decreased the levels of Aβ, APP, BACE 1, and hyperphosphorylated tau in 3×Tg-AD mice. However, the mechanism by which TBN inhibits Aβ deposition is still unclear. In this study, we employed APP/PS1 mice treated with TBN (60 mg/kg, ig, bid) for six months, and N2a/APP695swe cells treated with TBN (300 μM) to explore the mechanism of TBN in Aβ reduction. Our results indicate that TBN significantly alleviated cognitive impairment and reduced Aβ deposition in APP/PS1 mice. Further investigation of the underlying mechanisms revealed that TBN decreased the expression of APP and BACE1, activated the AMPK/mTOR/ULK1 autophagy pathway, inhibited the PI3K/AKT/mTOR/ULK1 autophagy pathway, and decreased the phosphorylation levels of JNK and ERK in APP/PS1 mice. Moreover, TBN was found to significantly reduce the mRNA levels of APP and BACE1, as well as those of SP1, CTCF, TGF-β, and NF-κB, transcription factors involved in regulating gene expression. Additionally, TBN was observed to decrease the level of *miR-346* and increase the levels of *miR-147* and *miR-106a* in the N2a/APP695swe cells. These findings indicate that TBN may reduce Aβ levels likely by reducing APP expression by regulating APP gene transcriptional factors and miRNAs, reducing BACE1 expression, and promoting autophagy activities.

## 1. Introduction

Alzheimer’s disease (AD) is an irreversible, progressive brain disorder that affects millions of individuals worldwide. It gradually destroys memory and cognitive abilities, as well as the ability to carry out simple tasks. The precise pathogenesis of AD remains unknown. However, it is characterized by changes in the brain, encompassing the formation of amyloid β (Aβ) plaques, the development of neurofibrillary tangles (NFTs), the onset of neuroinflammation, and the reduction in synaptic density. It is hypothesized that Aβ accumulation plays a central role in the pathogenesis of AD, acting as an initiating factor for subsequent pathological alterations, including the formation of NFTs, neuroinflammation, and synaptic loss [1]. Aβ is generated from amyloid precursor proteins (APPs) through two subsequent proteolytic cleavages by β-secretase and γ-secretase, which is known as the amyloidogenic pathway [2]. Conversely, the majority of APPs undergo non-amyloidogenic pathway cleavage by α-secretase, resulting in the production of sAPPα, which plays numerous neuroprotective roles in the maintenance of normal physiological function in neurons [3]. Normally, Aβ is degraded by proteasomes and Aβ-degrading enzymes (ADEs) in order to maintain the equilibrium between Aβ generation and clearance. However, in the pathological cases of AD, the level of APP expression is higher in the entorhinal cortex neurons of AD patients than in normal individuals [3]. Furthermore, the substrate affinity of β- and γ-secretases with APPs is increased, while the level of Aβ degradation is decreased, which leads to Aβ dyshomeostasis and the development of AD pathology [3]. Consequently, the adequate reduction in Aβ is considered to be the pivotal therapeutic objective of AD.

The strategies targeting the reduction in Aβ mainly include the inhibition of the amyloidogenic pathway, the promotion of the non-amyloidogenic pathway, the promotion of Aβ clearance, and the reduction in APP production [4]. In recent decades, the majority of pharmaceutical research into anti-amyloid therapy has focused on the development of secretase inhibitors and enhancers, as well as Aβ clearance agents, including the BACE1 inhibitor elenbecestat, the α-secretase enzyme enhancer APH1105, the Aβ aggregation inhibitor PBT1, and the Aβ monoclonal antibody lecanemab [5]. Lecanemab is the second FDA-approved immunotherapy for AD, which represents a significant advance in the ongoing efforts to develop effective treatments for AD. Apart from Aβ monoclonal antibody, autophagy is one of the important pathways involved in the cellular response for the removal of protein aggregates, including Aβ [6,7]. A substantial body of evidence indicates that autophagy enhancers such as rapamycin, lithium, carbamazepine, and latrepirdine protect against cognitive decline and inhibit Aβ aggregates in AD mouse models [6]. Nevertheless, the efficacy of autophagy stimulators in the treatment of AD patients requires further investigation through comprehensive clinical trials [6,8]. Therefore, it is important for researchers to explore additional strategies to combat Aβ.

In recent years, some researchers have attempted to identify a solution by inhibiting Aβ production from the perspective of APP gene expression levels [9]. It has been demonstrated that the number of copies of the APP gene, the level of APP mRNA, and the level of APP in single neuronal cells of sporadic AD are increased [10,11]. The process of APP gene transcription to APP mRNA is complicated, involving transcriptional and post-transcriptional regulation. Some of the APP translational modulators include 4-(5-methyl-1*H*-benzimidazol-2-yl) aniline (JTR-009), 2-[(pyridine-2-ylmethyl)-amino]-phenol (2-PMAP), and posiphen, which have been developed for the treatment of AD [12]. Posiphen, an inhibitor of the transcription of APP mRNA through the targeting of the iron-response element (IRE), has been demonstrated to reduce APP mRNA at the transcriptional level, having completed phase III clinical trials with mild to moderate AD [13]. Although the clinical data for posiphen have not yet been published, the inhibition of APP mRNA represents a promising avenue for the treatment of AD [4]. Recent advances in molecular biology have revealed that several microRNAs (miRNAs) regulate gene expression post-transcriptionally by binding mRNA targets and inhibiting translation or mRNA degradation [14]. These findings have identified miRNAs as a regulator of the expression of the APP gene, which is thought to be a causal factor in Aβ formation [9]. It has been demonstrated that *miR-106a*, *miR-135a*, *miR-153*, and *miR-147* bind directly to the 3′-untranslated region (UTR) of APP mRNA, which has been shown to downregulate APP expression at the post-transcriptional level. Conversely, evidence indicates that *miR-346* targets the 5′-UTR of APP mRNA, thereby promoting APP translation and Aβ production [15]. Nevertheless, growing numbers of miRNAs have been investigated for their potential role as inhibitors of the mRNA expression related to Aβ clearance pathways. Although there are some challenges to be addressed before miRNAs can be used in clinical practice, the potential of these small RNA molecules as therapeutic agents for AD is an area of active research [16,17]. In light of these considerations, it seems unlikely that a single pathway will prove effective in inhibiting Aβ generation or deposition. A promising strategy for reducing Aβ plaque generation, therefore, may be to combine multiple pathways, such as the inhibition of amyloidogenic secretases, the promotion of Aβ clearance, and the inhibition of APP production.

Tetramethylpyrazine nitrone (TBN; (2-[[(1,1-dimethylethyl) oxidoimino]-methyl]-3,5,6-trimethylpyrazine)) is a novel compound that has been equipped with a potent free radical scavenging nitrone moiety [18]. The therapeutic effects of TBN have been demonstrated in various animal models, including those of ischemic stroke, Parkinson’s disease (PD), amyotrophic lateral sclerosis (ALS), and AD [19,20,21,22,23]. Phase I and Phase II clinical trials of TBN for treatment of ischemic stroke, diabetic kidney disease, and ALS have been completed and have demonstrated promising results (CTR20190094, CTR20202126, CTR20210206, ChiCTR1900022848, ChiCTR2000039689, and ChiCTR2100043019) [24,25]. The results of a previous study in the 3×Tg-AD mouse model showed that TBN markedly improved cognitive function through a reduction in the Aβ levels and plaque deposition, tau hyperphosphorylation, and the restoration of synaptic function, which was likely associated with the inhibition of APP, BACE1, and PS1 protein expression [22]. Furthermore, TBN significantly inhibited the amyloidogenic processing pathway by inhibiting APP, BACE1, and PS1 expression and promoted the non-amyloidogenic processing pathway by increasing ADAM10 expression in N2a/APP695swe cells [22]. Additionally, the proteomic analysis of hippocampal neurons showed that TBN modulated the MAPK and mTOR pathways, both of which are involved in the regulation of autophagy. In order to provide further evidence of the efficacy of TBN for the treatment of AD, we conducted further evaluations of the anti-AD effects and the potential mechanisms of action in Aβ reduction by TBN in APP/PS1 mice in vivo and in N2a/APP695swe cells in vitro.

## 2. Results

### 2.1. TBN Improves Behavioral Performance in APP/PS1 Mice

**Figure 1:**
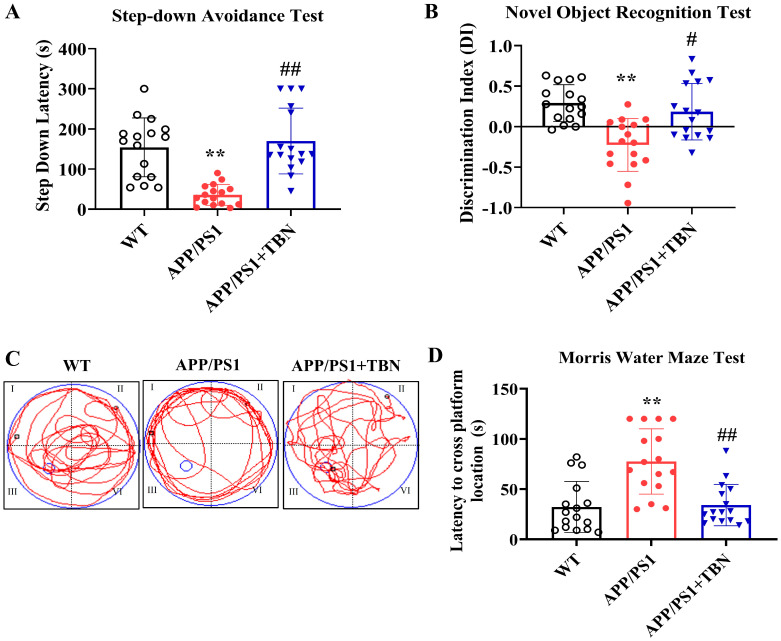
TBN improves cognitive abilities in APP/PS1 mice. (**A**) Latency of SDA test. (**B**) DI of NOR test. (**C**) MWM test during probe test. (**D**) Latency to cross platform location during probe test. Data are presented as means ± standard deviation (SD), with sample size of *n* = 16. The following symbols are used to indicate statistical significance: ** *p* < 0.01 vs. WT group and # *p* < 0.05 or ## *p* < 0.01 vs. APP/PS1 group. These symbols are used to indicate the results of one-way analysis of variance (ANOVA) followed by Tukey’s multiple comparison tests.

Cognitive improvement is an intuitive evaluation indicator of AD. In a series of behavioral experiments that can reflect long-term and short-term memory and learning ability, we tested whether TBN was capable of re-establishing cognitive function in APP/PS1 mice (Figure 1). TBN treatment resulted in a notable increase in latency in the step-down avoidance (SDA) test and a significant increase in the discrimination index (DI) in the novel object recognition (NOR) test when compared with the vehicle-treated APP/PS1 mice (Figure 1A,B). The results of the MWM test show that in comparison to the APP/PS1 group, the TBN-treated mice exhibited significantly shorter latency to locate the platform (Figure 1D), which had been removed, and a higher frequency of crossing the platform (Figure 1C). These findings are consistent with those previously reported in 3×Tg-AD mice [22]. Collectively, these results indicate that TBN is capable of restoring normal cognitive function in AD transgenic mouse models.

### 2.2. TBN Treatment Reduces Aβ Plaques in APP/PS1 Mice and Attenuates APP and BACE1 Expression in Hippocampus of APP/PS1 Mice

**Figure 2:**
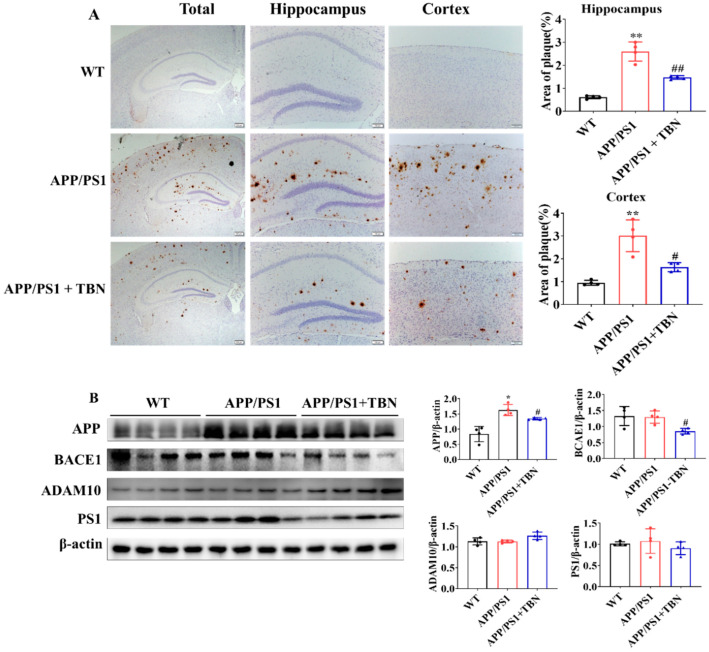
TBN decreases the Aβ plaques in APP/PS1 mice. (**A**) Aβ deposition was measured by using immunohistochemical staining with β-amyloid (D3D2N) antibody. The images were observed using a microscope at 10×, 20×, and 40× magnification. (**B**) Western blots and quantitative analyses of APP, BACE1, ADAM 10, and PS1 were conducted in the hippocampal tissues of the APP/PS 1 mice. The data are presented as means ± SD, with *n* = 4. Statistical significance was determined by using one-way ANOVA followed by Tukey’s multiple comparison tests. The following symbols are used to denote statistical significance: * *p* < 0.05 or ** *p* < 0.01 vs. WT group and # *p* < 0.05 or ## *p* < 0.01 vs. APP/PS1 group.

The deposit of Aβ plaques was identified by immunohistochemical staining. As illustrated in Figure 2A, the level of Aβ plaques exhibited a marked increase in the hippocampus and cortex of APP/PS1 mice and was significantly reduced following TBN treatment. It is known that Aβ is generated from APP via the amyloidogenic pathway. Here, we measured the protein expression of APP, BACE1, and PS1 in hippocampus. The results shown in Figure 2B indicate that the expression of APP and BACE1 was significantly decreased in the TBN-treated APP/PS1 mice in comparison with the saline-treated APP/PS1 mice. These findings are in accordance with those of a previous study conducted on 3×Tg-AD mice [22]. However, the expression of PS1 was slightly decreased by TBN treatment and showed no significant change, which differs from the result observed in 3×Tg-AD mice [22]. Moreover, the level of α-secretase, the principal secretase in the non-amyloidogenic pathway, was evaluated. The result shows that TBN increased the expression of ADAM10 without a significant difference. These results demonstrate that TBN can reduce the expression of APP and BACE 1 in both APP/PS 1 mice and 3×Tg-AD mice.

### 2.3. TBN Treatment Restores Levels of Synapse Proteins in Hippocampus of APP/PS 1 Mice

**Figure 3:**
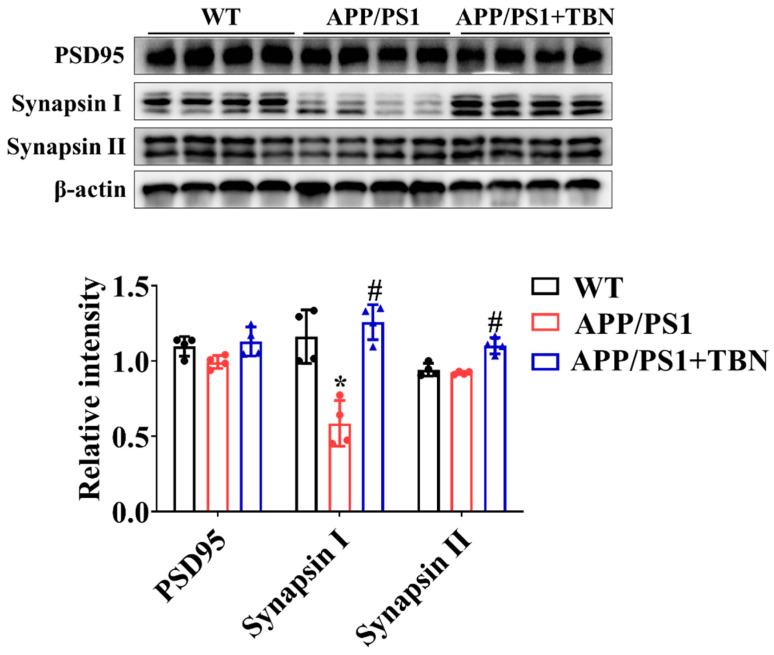
TBN affects the expression of synapse protein in the hippocampal tissues of APP/PS 1 mice. The levels of PSD 95, synapsin I, and synapsin II were measured by WB. The data are presented as means ± SD, with *n* = 4. Statistical significance was determined by using one-way ANOVA followed by Tukey’s multiple comparison tests. The following symbols are used to denote statistical significance: * *p* < 0.05 vs. WT group and # *p* < 0.05 vs. APP/PS1 group.

The levels of PSD 95, synapsin I, and synapsin II were measured by Western blotting. As compared with the saline-treated group, the expression of synapsin I and synapsin II was significantly increased after TBN treatment (Figure 3), indicating the protective effects of TBN on synaptic function. The results are consistent with those of a previous study in 3×Tg-AD mice [22].

### 2.4. TBN Enhances Aβ Clearance by Regulating Expression of Autophagy-Related Proteins in APP/PS1 Mice

**Figure 4:**
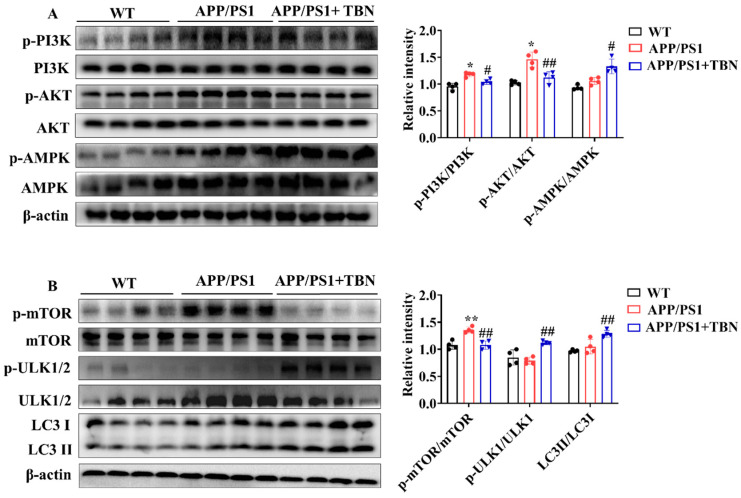
TBN enhances the expression of autophagy-related proteins in the hippocampal tissues of APP/PS 1 mice. (**A**,**B**) Representative images and quantitative analyses of the proteins p-PI3K, PI3K, p-AKT, AKT, p-AMPK, AMPK, p-mTOR, mTOR, p-ULK1/2, ULK1/2, LC3 I, and LC3 II. The data are presented as means ± SD, with *n* = 4. Statistical significance was determined by using one-way ANOVA followed by Tukey’s multiple comparison tests. The following symbols are used to denote statistical significance: * *p* < 0.05 or ** *p* < 0.01 vs. WT group and # *p* < 0.05 or ## *p* < 0.01 vs. APP/PS1 group.

The above results indicate that TBN can decrease Aβ production by inhibiting the expression of APP and BACE 1. However, it remains unclear whether TBN has other ways to reduce Aβ aggregation. It was previously established that the metabolism of Aβ is crucially influenced by autophagy. Consequently, the regulatory effects of TBN on autophagy were evaluated by detecting the expression of the key regulators involved in the autophagy pathway. AMPK activation has been demonstrated to suppress the activation of mTOR, thereby promoting the process of the autophagy pathway. Furthermore, AMPK activation has also been shown to stimulate ULK-mediated autophagy pathway initiation. In addition to AMPK, the upstream kinase of mTOR, PI3K/AKT kinase suppression can also stimulate the autophagy pathway. The results presented in Figure 4A demonstrate that TBN treatment led to a notable increase in the ratio of p-AMPK/AMPK and a decrease in the ratios of p-PI3K/PI3K and p-AKT/AKT, compared with the saline-treated APP/PS1 mice. Further investigation revealed that TBN treatment resulted in a reduction in the ratio of p-mTOR/mTOR and an increase in the ratios of p-ULK1/ULK1 and LC3II/LC3I. This suggests that the enhancement effect of TBN on Aβ reduction is also mediated by autophagy via the activation of the AMPK/mTOR/ULK1 pathway and the inhibition of the PI3K/AKT/mTOR/ULK 1 pathway.

### 2.5. TBN Reduced Transcription of APP Gene by Regulating Transcription Factors and miRNA Levels

**Figure 5:**
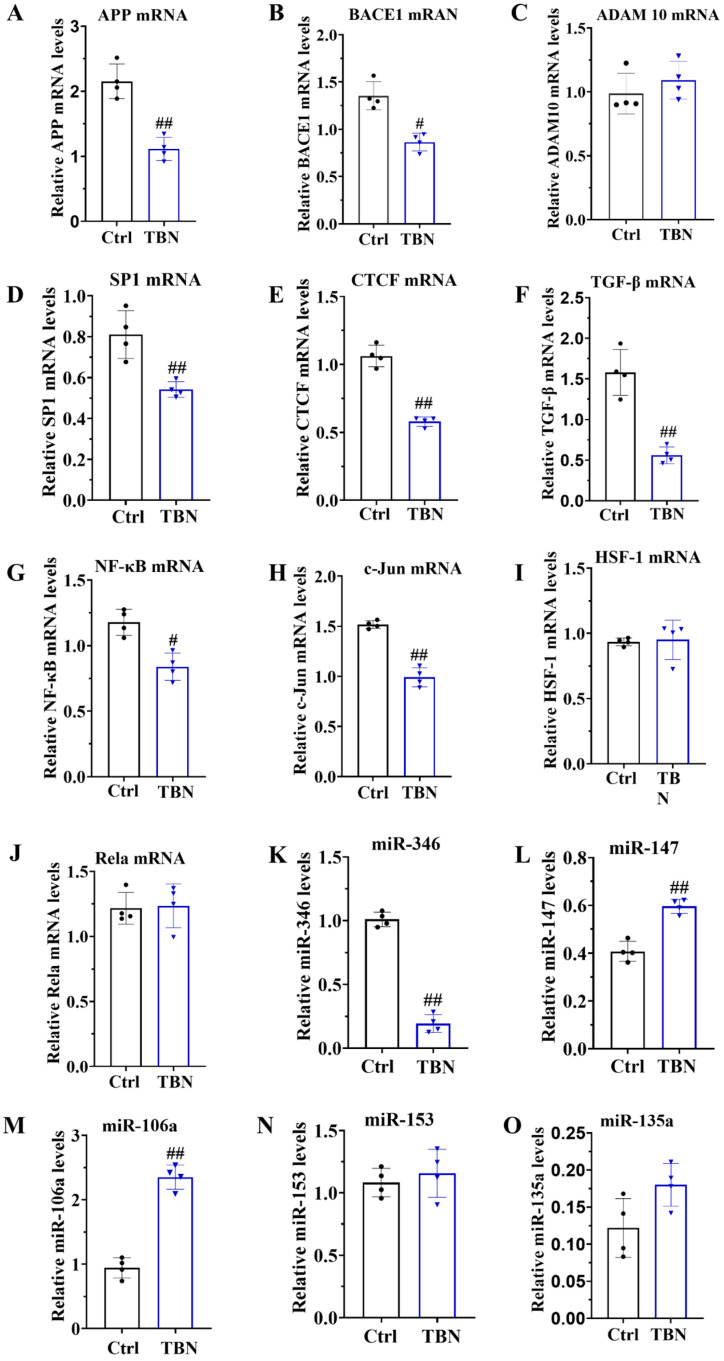
TBN affects the modulation of APP mRNA in N2a/APP695swe cells. N2a/APP695swe cells were treated with or without TBN (300 μm) for 24 h, and the levels of mRNA and miRNA were detected by quantitative reverse transcriptase PCR. (**A**–**C**) Representative quantitative analyses of the APP mRNA, BACE1 mRNA and ADAM 10 mRNA. (**D**–**J**) Representative quantitative analyses of the SP1 mRNA, CTCF mRNA, TGF-β mRNA, NF-κB mRNA, c-Jun mRNA, HSF-1 mRNA and Rela mRNA. (**K**–**O**) Representative quantitative analyses of the *miR-346*, *miR-147*, *miR-106a*, *miR-153* and *miR-135a*. The data are presented as means ± SD, with *n* = 4. The statistical significance of the differences between the groups was determined by using an unpaired, two-tailed *t*-test, with a *p*-value of # *p* < 0.05 or ## *p* < 0.01 indicating a statistically significant difference when compared with the APP/PS1 group.

As demonstrated in Figure 1B, TBN can reduce the level of APP expression. However, the mechanism by which TBN affects APP reduction remains unclear. In order to gain further insights, we quantified the levels of APP mRNA, BACE1 mRNA, and ADAM 10 mRNA by qRT-PCR in N2a/APP695swe cells. The results, presented in Figure 5A,B, indicate that TBN treatment significantly decreased the APP mRNA and BACE 1 mRNA transcription levels compared with the control group. TBN treatment also exhibited a slight increase in ADAM 10 mRNA levels, although this was not statistically significant compared with the control group (Figure 5C).

To gain further insights into the underlying mechanisms of TBN in causing a reduction in APP mRNA expression, the transcription factors that promote APP transcription were quantified in the N2a/APP695swe cells by using qRT-PCR. These included the following transcription factors: activator protein-1 (AP-1), stimulating protein 1 (SP-1), CCCTC-binding factor (CTCF), transforming growth factor-β (TGF-β), heat shock transcription factor 1 (HSF-1), c-Jun, and nuclear factor (NF)-κB/Rela. The results, presented in Figure 5D–J, indicate that the mRNA levels of SP1, CTCF, TGF-β, NF-κB, and c-Jun were significantly reduced by TBN treatment, while the levels of HSF-1 and Rela mRNA remained unaltered. These findings indicate that TBN can downregulate APP mRNA levels by inhibiting the expression of transcriptional factors. Furthermore, the levels of *miR-346*, *miR-147*, *miR-106a*, *miR-153*, and *miR-135a* were quantified by using qRT-PCR in the N2a/APP695swe cells. The results presented in Figure 5K–O indicate that TBN treatment significantly decreased the level of *miR-346* while simultaneously increasing the levels of *miR-147* and *miR-106a* compared with the control cells. TBN treatment also increased the levels of *miR-153* and *miR-135a*, although these changes were not statistically significant. These findings suggest that TBN can inhibit APP translation by regulating miRNA levels.

**Figure 6:**
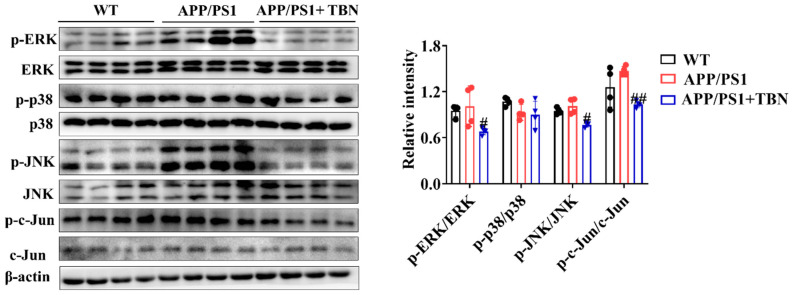
The effects of TBN on the MAPK signaling pathway. Representative images and quantitative analyses of the proteins p-ERK, ERK, p-p38, p38, p-JNK, JNK, p-c-Jun, and c-Jun. The data are presented as means ± SD, with *n* = 4. Statistical significance was determined by using one-way ANOVA followed by Tukey’s multiple comparison tests. The following symbols are used to denote statistical significance: # *p* < 0.05 or ## *p* < 0.01 vs. APP/PS1 group.

In addition, the results of the investigation displayed in Figure 6 indicate that TBN treatment could reduce the ratios of p-ERK/ERK, p-p38/p38, p-JNK/JNK, and p-c-Jun/c-Jun in the hippocampus of APP/PS 1 when compared with the saline-treated mice. It is known that c-Jun is a component of AP-1, a transcription factor that exerts a direct influence on APP promoter activity. Moreover, AP-1 is activated and regulated by MAP kinases (MAPKs). These results suggest that TBN may reduce AP-1 activity by inhibiting the phosphorylation level of MAPKs, which in turn leads to the downregulation of APP mRNA.

In conclusion, TBN was found to reduce APP expression by regulating the transcriptional factors involved in APP mRNA transcription, including AP-1, SP1, CTCF, TGF-β, and NF-κB. Concurrently, TBN acts on the post-transcriptional regulation of APP by decreasing miRNA levels.

## 3. Discussion

In our previous study, we demonstrated that TBN significantly reduced Aβ production and deposition in 3×Tg-AD mice [22]. The present study aimed to further evaluate the therapeutic effects of TBN in the APP/PS1 mice model. Our findings demonstrate that TBN also significantly reduced Aβ production and deposition in APP/PS1 mice. The mechanism by which TBN reduces Aβ levels is likely through the inhibition of APP expression via the modulation of APP gene transcriptional factors and miRNAs, the inhibition of BACE 1 expression, and the promotion of autophagy activities.

APP is a type I single-pass transmembrane protein that is the direct source of Aβ generation. Treating AD by targeting APP mRNA with a small-molecule compound is becoming a popular idea because lowering the steady-state level of its precursor protein effectively suppresses Aβ production [12]. The regulation of APP gene expression is a complex process involving a number of different factors. These include the gene promoter, which contains a high GC region with five GGGCGC boxes, as well as transcription factors such as SP-1, AP-1, CTCF, TGF-β, HSF-1, and NF-κB; growth factors such as nerve growth factor; cytokines such as interleukin-1; and post-transcriptional regulators such as miRNA. SP-1, AP-1, CTCF, TGF-β, HSF-1, and NF-κB facilitate transcription in the promotor of the APP gene [9]. Our studies demonstrated that TBN significantly decreases the levels of SP-1 mRNA, c-Jun mRNA, TGF-β mRNA, and NF-κB mRNA in N2a/APP695swe cells. This suggests that TBN reduces APP mRNA by modulating the levels of related transcription factors. Of note, TBN also decreases p-c-Jun expression in the hippocampus of APP/PS1 mice. C-Jun is the subunit of AP-1. AP-1 is a dimeric protein composed of c-Fos, c-Jun, and related proteins which effectively promotes APP gene transcription to mRNA. This process is initiated by the phosphorylation of MAPKs, which are known to regulate a number of key cellular processes, including gene expression, proliferation, apoptosis, and inflammation. These processes are involved in the pathophysiology and pathogenesis of AD [26]. Three main distinct subgroups of MAPKs have been identified in mammalian cells, namely, extracellular-regulated kinase (ERK), c-Jun N-terminal kinase (JNK), and p38. These three pathways are activated in vulnerable neurons in patients with AD [26,27]. ERK regulates the expression of c-Fos, whereas JNK exerts precise control over the levels of c-Jun. Additionally, p38 modulates the activity of p53 and ATF2 [28]. A substantial body of research has demonstrated that the inhibition of ERK, p38, or JNK restores spatial memory and short-term and long-term memory and reduces the number of Aβ deposits [26,29,30,31]. Furthermore, MAPKs not only regulate APP mRNA transcription but also regulate tau phosphorylation [26]. These viewpoints are consistent with the results of our study, which can explain the mechanism of TBN in APP mRNA reduction. It can be postulated that TBN inhibits the phosphorylation levels of JNK and ERK, thereby reducing the level of p-c-Jun, which in turn leads to a reduction in AP-1 and finally to a reduction in APP mRNA. Furthermore, our previous study indicated that TBN can reduce tau hyperphosphorylation in 3×Tg-AD mice. This may be associated with the inhibition of the MAPK signaling pathway by TBN.

Another significant finding is that TBN can reduce the level of *miR-346* and increase the levels of *miR-147* and *miR-106a* in N2a/APP695swe cells. microRNAs are small, non-coding RNA molecules that play a pivotal role in regulating gene expression or mRNA degradation at the post-transcriptional level. They influence various processes associated with AD, including Aβ aggregation, tau pathology, mitochondrial dysfunction, neuroinflammation, and synaptic dysfunction [32]. Recently, miRNAs presented a promising, cost-effective, and non-invasive class of biomarkers for AD [33]. Some miRNAs affect Aβ metabolism by controlling the expression of APP and the enzymes that process it. For example, *miR-106a*, *miR-135*, *miR-147*, *miR-101*, *miR-16*, and *miR-520c* bind to the APP 3‘-UTR and negatively regulate reporter gene expression. The over-expression of these miRNAs results in reduced APP protein levels [14]. Liang et al. have demonstrated that miR-153 suppresses the expression of APP by specifically targeting the site within the APP 3′-UTR [34]. In contrast, miR-346 has been demonstrated to target the APP mRNA 5′-UTR, thereby upregulating APP translation and Aβ production [35]. miRNAs can also affect AD pathology in other ways. It has been demonstrated that the expression of BACE 1 can be increased by a number of miRNA, including *miR-29a/b-1*, *miR-107*, *miR-298*, *miR-124*, *miR-135b*, *miR-188*, and *miR-338* [33]. Both *miR-34a* and *miR-26b* have been demonstrated to inhibit tau expression and affect NFT formation [36]. Furthermore, *miR-499* and *miR-30* have been demonstrated to regulate mitochondrial mobility within the cell by targeting proteins involved in the processes of fission and fusion [32]. Additionally, studies have demonstrated that *miR-26b*, *miR-132*, and *miR-206* can target neurotrophic factors to regulate synaptic function [36]. Moreover, recent studies have demonstrated that miRNAs modulate autophagic activity. For instance, the gene of mTOR was regulated by several miRNAs, including *miR-100*, *miR-96*, *miR-338-3p*, *miR-7*, and *miR-128*. Additionally, ULK 1 expression can be suppressed by *miR-290/295* [37]. Our findings also indicate that TBN significantly reduced the BACE 1 mRNA levels in N2a/APP695swe cells and activated autophagy by decreasing mTOR and increasing ULK expression in the hippocampus of APP/PS1 mice. These effects may be related to the regulation of miRNAs by TBN, which requires further investigation.

In conclusion, this study represents a preliminary investigation into the potential mechanism of TBN in reducing APP levels. It has been demonstrated that TBN exerts a novel regulatory effect on miRNAs. However, a considerable degree of validation work remains to be performed before these findings can be validated. This includes the verification of the impact of TBN on miRNAs in the hippocampus of APP/PS1 mice, as well as the investigation of whether TBN affects other miRNAs, such as BACE 1, as well as autophagy, and synaptic function. Despite the approval of two antibody-based drugs, aducanumab and lecanemab, by the FDA, there remains a necessity for the development of safe and efficacious pharmacological treatments capable of halting or at the very least postponing the progression of AD. TBN treatment has been demonstrated to reverse the learning and memory deficits observed in AD mice; therefore, TBN is a promising drug candidate for AD treatment.

## 4. Materials and Methods

### 4.1. Chemicals and Reagents

TBN was synthesized by Guangzhou Magpie Pharmaceuticals Co., Ltd. (Guangzhou, China). DMEM, FBS, G418, and penicillin–streptomycin solution (100×) were purchased from Gibco^TM^ (Waltham, MA, USA). RIPA buffer and protease/phosphatase inhibitor cocktail were purchased from Thermo Fisher Scientific (Waltham, MA, USA). Antibody APP, BACE1, synapsin I, ADAM10, synapsin II, PSD 95, PS1, and goat anti-mouse IgG H + L (Alexa Fluor^®^ 488) were purchased from Abcam (Cambridge, UK). Antibody D3D2N, p-AMPK, AMPK, p-PI3K, PI3K, p-AKT, AKT, p-mTOR, mTOR, p-ULK1/2, ULK1/2, LC3, p-ERK, ERK, p-p38, p38, p-JNK, JNK, p-c-Jun, c-Jun, β-actin, anti-rabbit IgG, HRP-linked antibody, and anti-mouse IgG antibody were bought from Cell Signaling Technology (Beverly, MA, United States). Pure Nitrocellulose Blotting Membrane (66485) was obtained from PALL Corporation (New York, NY, USA). TRleasy^TM^ LS Total RNA Extraction Reagent (19201ES60), Hieff qPCR SYBR Green Master Mix (no ROX) (11201ES08), and Hifair III 1st Strand cDNA Synthesis SuperMix for qPCR (11141ES10) were purchased from YEASEN Biotechnology (Shanghai, China). SDA, NOR, and MWM devices and software were bought from Huaibei Zhenghua Biological Instrument Equipment Co., Ltd. (Huaibei, Anhui, China).

### 4.2. Cell Culture

N2a/APP695swe cells (N2a cells stably transfected with the human APP gene) were kindly donated by Dr. Xifei Yang’s lab (Key Laboratory of Modern Toxicology of Shenzhen, Shenzhen Center for Disease Control and Prevention, Shenzhen, China). N2a/APP695swe cells were cultured in DMDM with 10% FBS, penicillin–streptomycin (1×), and 200 μg/mL G418 at 37 °C in 95% air and 5% CO_2_ in a humidified incubator. The cells were inoculated in 25 cm^2^ culture flasks at a specified density for 24 h, after which they were treated with or without 300 μM TBN. After a further 24 h, the cells were collected for RT-PCR, which was measured according to the instructions of the kits.

### 4.3. Animal Care and Treatment

APP/PS1 mice were purchased from the Jackson Laboratory and subsequently inbred in the laboratory animal center of the Shenzhen CDC. The mice were housed under controlled conditions, with a temperature range of 20~26 °C, humidity of 40~70%, and 12 h light/12 h dark cycles. APP/PS1 mice are hemizygous mice expressing human APP and a mutant human PS1-dE9, and their genotype was confirmed by polymerase chain reaction on a sample of the tail. The littermates of wild-type mice served as the negative control group (WT group, *n* = 16, half female). Two-month-old APP/PS1 mice were randomly allocated to the vehicle group (APP/PS1 group) and the TBN group (*n* = 16, half female). The mice in the TBN group were treated with TBN at a dose of 60 mg/kg via intragastric administration twice daily. The WT and APP/PS1 group mice received a parallel volume of saline. Following a six-month period of TBN or saline administration, all mice underwent a behavioral assessment, after which they were sacrificed via the injection of anesthetics (Zoletil 50/xylazine).

### 4.4. Behavioral Tests

Behavioral tests commenced at eight months of age and continued for approximately four weeks, throughout which the treatment was maintained. The SDA test, NOR test, and MWM test were performed in accordance with the methodology previously described [22,38,39]. In brief, the SDA test was performed by placing the mouse in a separate compartment with a grid floor underneath and an insulated wooden platform. During the training period, the mouse was placed on the grid floor, and electrical shock was delivered for a period of 5 min. In the testing period, the mouse was placed on the wooden platform while electricity was applied. Step-down latency is defined as the time taken for the mouse to make contact with the grid floor for the first time. The error number is defined as the number of times the grid floor was touched by the mouse. The NOR test was conducted on the fourth day following the SDA test. For the NOR test, the mouse was placed in an open field with two identical objects and permitted to explore freely for a period of 5 min once daily for 3 days during the training phase. On the fourth day, the mouse was placed in the open field where an old object was replaced with a new object, and its exploration trajectory was recorded by video-tracking software. The discrimination index (DI), which represents the ability to recognize new objects, was calculated by using the following equation: (Time exploring new object − Time exploring old object)/(Time exploring new object + Time exploring old object) × 100. Similarly, MWM was performed on the fourth day following the NOR test. The NOR test was carried out in a circular tank with a platform in the third quadrant and filled with opaque water. During the training phase, the mouse was trained to find the platform four times a day for 5 days. A probe trial was conducted on the sixth day following the training session. During this trial, the platform was removed, and the mouse was placed in the tank to explore the platform site. The mouse’s swimming trajectory was recorded by a movement-tracking system (Super Maze^+^ software V 2.0). The latency to cross platform location was defined as the time taken for the mouse to cross the platform site for the first time.

### 4.5. Immunohistochemistry

The Aβ plaques in the hippocampus and cerebral cortex were quantified by using the D3D2N antibody by immunohistochemical staining, in accordance with the methodology previously described [22,38]. In brief, the brain tissues were subjected to a series of processes, including fixation in 4% paraformaldehyde, embedding in paraffin, sectioning, dewaxing with xylene, gradual rehydration with ethanol, antigen retrieval, peroxidase removal by 3% hydrogen peroxide, blocking with 10% FBS, primary antibody and secondary antibody incubation, diaminobenzidine color development, and polyvinylpyrrolidone mounting. To obtain digital images of the sections, an Olympus BX41 microscope was employed.

### 4.6. Western Blotting

The Western blotting procedure was conducted in accordance with the methodology previously described in [22,38]. Briefly, hippocampus tissues were homogenized in RIPA lysis buffer containing PMSF and then centrifuged to obtain the supernatant. Subsequently, the protein concentration was determined, after which the proteins were subjected to SDS-PAGE by using either 4% or 12% gels. The proteins were transferred to a nitrocellulose (NC) blotting membrane after electrophoresis. The NC membrane was then washed for 5 min with Tris-buffered saline with Tween 20 (TBST) and blocked with TBST buffer containing 5% non-fat dry milk for 1 h at room temperature. The membrane was washed with TBST for 3 times and incubated with the primary antibody overnight at 4 °C. The membrane was incubated with the secondary antibody for 1 h at room temperature. Finally, the blot was scanned by a chemiluminescent imaging system (Tanon 5200 Multi) (Shanghai, China), and the bands were quantified with densitometry by using Image J (Version 2).

### 4.7. qRT-PCR

The total RNA was extracted from the N2a/APP695swe cells by using TRleasy^TM^ LS Total RNA Extraction Reagent, in accordance with the manufacturer’s instructions. Quantitative PCR was conducted by a real-time system (KUBO TECH, q225) using Hifair III 1st Strand cDNA Synthesis SuperMix for qPCR kits and Hieff qPCR SYBR Green Master Mix kit (no ROX) in accordance with the manufacturer’s instructions. The results of the qPCR were normalized by using GAPDH for mRNA and U6 for miRNA. The primers used for qRT-PCR are listed below: *APP*: forward, 5′-CCAGAACTGGTGCAAGCG-3′; reverse, 5′-TTTGGCGACGGTGTGCCA-3′. *BACE1*: forward, 5′-TGTGGAGATGGTGGACAACC-3′; reverse, 5′-GGACAGCTGCCTCTGGTAG-3′. *ADAM 10*: forward, 5′-AGTAGAGGAAGGAGCCCGG-3′; reverse, 5′-GCAATCACAGCTTCTCGTGT-3′. *SP1*: forward, 5′-ATGCTGCTCAACTCTCCTCCATG-3′; reverse, 5′-CCTTCTCCACCTGCTGTCTCATC-3′. *CTCF:* forward, 5′-TTGAGGAAGAACAGCAGGAAGGAC-3′; reverse, 5′-TGGCTTCGGAGGCTTCATATTACC-3′. *TGF-β:* forward, 5′-GGGCATCGCTCATCTCCACAG-3′; reverse, 5′-GCAACAGGTCAAGTCGTTCTTCAC-3′. *NF-κB:* forward, 5′-ATGGGAAACCGTATGAGCCTGTG-3′; reverse, 5′-AGTTGTAGCCTCGTGTCTTCTGTC-3′. *c-Jun*: forward, 5′-GTTGCGGCCGCGAAACTT-3′; reverse, 5′-CATTGCCCTCGAGCCCTG-3′. *HSF-1:* forward, 5′-CCAAGGAGGTGCTGCCCAAG-3′; reverse, 5′-ATGCTGGAACTCGGTGTCATCTC-3′. *Rela:* forward, 5′-AGAGAAGCACAGATACCACCAAGAC-3′; reverse, 5′-GGTCAGCCTCATAGTAGCCATCC-3′. *GAPDH*: forward, 5′-TCCACTCACGGCAAATTCAAC-3′; reverse, 5′-GTAGACTCCACGACATACTCAGC-3′. *miR-346*: forward, 5′-ACACTCCAGCTGGGTGTCTGCCCGAGTGC-3′; reverse, 5′-CTCAACTGGTGTCGTGGA-3′. *miR-147*: forward, 5′-ACACTCCAGCTGGGTGGAAACTTTCTGCA-3′; reverse, 5′-CTCAACTGGTGTCGTGGA-3′. *miR-106a*: forward, 5′-ACACTCCAGCTGGGCAAAGTGCTAACAGT-3′; reverse, 5′-CTCAACTGGTGTCGTGGA-3′. *miR-153*: forward, 5′-ACACTCCAGCTGGGTTGCATAGTCACAA-3′; reverse, 5′-CTCAACTGGTGTCGTGGA-3′. *miR-135a*: forward, 5′-ACACTCCAGCTGGGTATAGGGATTGGAGCC-3′; reverse, 5′-CTCAACTGGTGTCGTGGA-3′.

## 5. Statistical Analysis

Quantitative data are presented as means ± SD. Statistical significance was determined by using one-way ANOVA followed by Tukey’s multiple comparison tests or an unpaired, two-tailed *t*-test. A *p*-value below 0.05 was significant. 

## Data Availability

The original contributions presented in the study are included in the article, further inquiries can be directed to the corresponding authors.
